# Integrative ATAC-Seq and RNA-Seq Analysis Reveals Key Transcription Factors Mediating Low Salinity Adaptation in Penaeid Shrimp

**DOI:** 10.3390/ijms26104605

**Published:** 2025-05-11

**Authors:** Chuntao Zhang, Jianbo Yuan, Roujing Li, Zhanyuan Yang, Man Luo, Xiaoyun Zhong, Jie Hu, Shuqing Si, Xiaojun Zhang, Fuhua Li

**Affiliations:** 1 Key Laboratory of Breeding Biotechnology and Sustainable Aquaculture (CAS), Institute of Oceanology, Chinese Academy of Sciences, Qingdao 266500, China; zct486855@163.com (C.Z.); ytroujingl@163.com (R.L.); yzyqdu@163.com (Z.Y.); luoman24@mails.ucas.ac.cn (M.L.); zhongxiaoyun5681@163.com (X.Z.); hujie543@163.com (J.H.); m17862667253@163.com (S.S.); xjzhang@qdio.ac.cn (X.Z.); fhli@qdio.ac.cn (F.L.); 2 University of Chinese Academy of Sciences, Beijing 101400, China; 3 Laboratory for Marine Biology and Biotechnology, Qingdao Marine Science and Technology Center, Qingdao 266071, China; 4 Shandong Province Key Laboratory of Experimental Marine Biology, Institute of Oceanology, Chinese Academy of Sciences, Qingdao 266500, China

**Keywords:** penaeid shrimp, RNA-seq, ATAC-seq, transcription factor, low salinity

## Abstract

Salinity serves as an important environmental factor in ecosystems, driving the evolution of adaptive strategies in euryhaline species. The Pacific white shrimp, *Litopenaeus vannamei*, is a representative euryhaline species. However, the molecular mechanisms, particularly the roles of cis-regulatory elements, remain elusive in penaeid shrimp. This study tackles this gap by subjecting *L. vannamei* to a gradual reduction in salinity from 30‰ to 3‰, and then applying ATAC-seq and RNA-seq techniques to dissect the cis-regulation mechanisms underlying low salinity adaptation. A key finding reveals a positive correlation between chromatin accessibility and gene expression, with 36.8% of differentially expressed genes directly associated with changes in chromatin accessibility. The cis-regulation of many osmoregulation-related pathways, such as betaine synthesis and PI3K-Akt signaling pathways, appeared to be a crucial strategy for salinity adaptation in shrimp. By analyzing differentially accessible regions under low salinity stress, we uncovered two known and seven novel candidate transcription factors (TFs) that may play pivotal roles in salinity adaptation. We further conducted a comprehensive analysis of these TFs, including their functions, expression profiles, consensus TFBS motifs, and the functional enrichment and expression profiles of their targeted genes. This study elucidates a complex cis-regulatory network that enables exceptional salinity tolerance in *L. vannamei*, which provides a foundation for the refinement of genetic breeding programs and desalination aquaculture for penaeid shrimp.

## 1. Introduction

Shrimp are among the most economically important marine species, and *Litopenaeus vannamei* is the most valuable single shrimp species globally. As an euryhaline organism, penaeid shrimp exhibit remarkable adaptability to a wide salinity range [1]. They can inhabit environments ranging from the low salinity of brackish water (1‰) to the highly saline conditions of saltwater (40‰) [2,3]. Salinity is a crucial environmental factor that significantly influences key aspects of shrimp physiology, including growth, respiration, metabolism, survival, osmotic balance, immunity, and oxidative functions [4]. Exposure to varying salinity levels triggers adaptive physiological responses that may lead to functional deviations, and ultimately affect growth performance and survival rates [5]. In recent decades, *L. vannamei* has become a globally significant aquaculture species particularly due to its remarkable adaptability to a wide range of salinity conditions. However, despite these achievements, questions regarding its osmoregulatory strategies and the variability in its low salinity tolerance persist.

Penaeid shrimp have evolved multiple adaptive mechanisms to cope with salinity fluctuations, including the modulation of energy consumption [1,5], regulation of antioxidant enzyme activity [6], body immunity [7], and osmoregulation gene expression [8]. The adaptation of penaeid shrimp to low-salinity environments involves complex gene regulatory networks. However, limited research has focused on the upstream cis-regulatory elements and signaling pathways involved in this process. Consequently, the regulatory network and the pivotal osmoregulation transcription factors (TFs) involved in low salinity adaptation remain elusive in penaeid shrimp.

Active cis-regulatory elements are typically located in open chromatin regions without nucleosomes and are able to bind proteins, such as TFs. These TFs can be recognized and play roles in certain development and differentiation processes [9]. Several TFs were identified to play important roles in salinity adaptation, including NRF2, CREB, OREBP, HIF-α, and NAC. The transcription factor NRF2 plays an osmoregulatory role in the brain of *Coilia nasus* in coordination with AQP1 to stimulate the activity of downstream antioxidant enzymes to resist salinity stress [10]. The HIF gene plays a role in regulating molting and immune response under different salinity conditions, particularly in tissues like the gills and hepatopancreas in *Metacarcinus magister* [11]. CREB can coordinate with cyclic adenylate (cAMP) and protein kinase (PKA) to increase *Na^+^-K^+^-ATPase* (NKA) activity under the stimulation of low salt stress and bioamines in the gill of *L. vannamei* [12]. To date, these TFs have not been investigated in penaeid shrimp. Furthermore, as a typical euryhaline species, *L. vannamei* may adopt special osmoregulation mechanisms and some undiscovered unique TFs.

The dynamic transition between repressive and active chromatin states represents a crucial regulatory mechanism that enables shrimp to adapt to fluctuating environmental conditions. Open chromatin facilitates the binding of TFs at the transcriptional level. The Assay for Transposase Accessible Chromatin with High-Throughput Sequencing (ATAC-seq) is a powerful chromatin accessibility profiling technique that enables the genome-wide identification of regulatory elements, including promoters, enhancers, and transcription factor binding sites [13,14]. The integrative analysis of ATAC-seq and RNA-seq can help resolve the upstream regulatory mechanisms of gene expression and discover new gene regulatory interactions.

In this study, we adopted an integrated ATAC-seq and RNA-seq approach to explore the cis-regulatory mechanisms underlying low salinity adaptation in penaeid shrimp and identified key promoter regions and their associated genes involved in salinity adaptation. Nine putative functional TFs and their binding sites (TFBSs) associated with salinity adaptation were identified by analyzing differentially open chromatin regions. We performed comprehensive analyses of these TFs, conserved motifs of TFBSs, functional enrichment results of target genes, and their expression profiles. Our findings provide novel insights into the cis-regulatory mechanisms underlying low salinity adaptation in penaeid shrimp, establishing a foundation for future genetic improvement programs and the development of sustainable low-salinity aquaculture practices.

## 2. Results

### 2.1. Differentially Expressed Genes in Shrimp in Response to Low Salinity Stress

**Figure 1:**
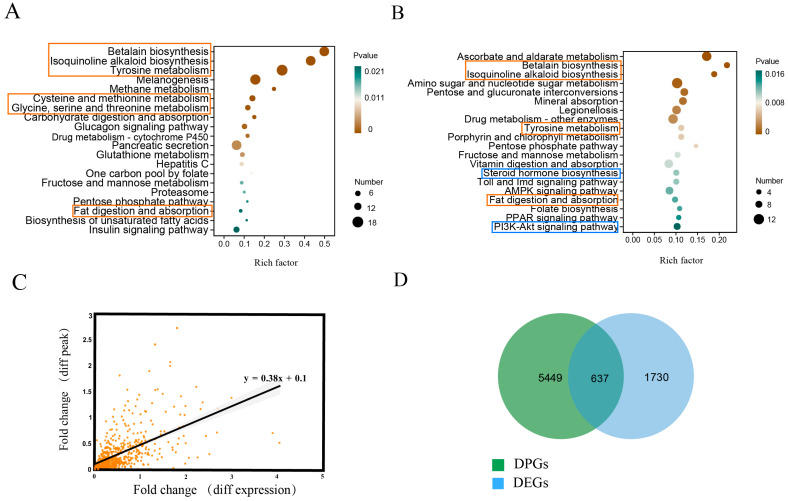
Integrated analysis of RNA-seq and ATAC-seq in penaeid shrimp under low salinity stress: (**A**) KEGG enrichment analysis of the DEGs in penaeid shrimp under low salinity stress. (**B**) KEGG enrichment analysis of the DEGs with differentially chromatin accessibilities (DE-DPGs) in shrimp under low salinity stress. The orange rectangle indicates pathways co-enriched in (**A**,**B**), while the blue one denotes salinity-specific pathways exclusive to (**B**). (**C**) Correlation analysis between the fold changes in gene expression and fold changes in the normalized read density values of DPs in the ATAC-seq analysis. (**D**) The intersection of the DP-related genes (DPGs) and DEGs.

RNA-seq analysis helps identify potential osmoregulation mechanisms triggered by low salinity conditions and also identifies differentially expressed genes (DEGs) that may be regulated by the regulatory purview of TFs. Hepatopancreas samples of *L. vannamei* with salinities of 30‰ (control group), 9‰, and 3‰ (low salinity stress groups) were collected for RNA-seq and differential gene expression analyses. Among them, 189 differentially expressed genes (DEGs) were identified between 30‰ and 9‰ salinity conditions, 393 DEGs between 30‰ and 3‰, and 180 DEGs between 3‰ and 9‰. After removing the overlapping items among these identified DEGs, a total of 514 DEGs (fold change > 2 and *FDR q*-value < 0.05) were identified in shrimp under low salinity stress. These DEGs showed functional enrichment in several critical pathways integral to salinity adaptation, including free amino acid metabolism, lipid metabolism, and betaine synthesis (Figure 1A). Notably, several key osmoregulation genes (including NKA, NKCC, and V-ATPase) and stress-responsive genes (including HSP and CYP450) also exhibited differential expression patterns (Supplementary Materials Figures S3–S7).

Both free amino acid and lipid metabolism were crucial for salinity acclimation. Many pathways associated with free amino acid and lipid metabolism were differentially regulated, such as the glycine, serine, and threonine metabolic pathway; the cysteine and methionine metabolic pathway; the tyrosine metabolic pathway; and the fatty acid metabolic pathway (Figure 1A). A total of 41 DEGs involved in amino acid metabolism and 20 DEGs involved in lipid metabolism were identified, and most of them were upregulated in response to the decreasing salinity (Supplementary Materials Figures S1 and S2).

Glutathione S-transferase (GST), heat shock proteins (HSPs), and cytochrome P450 (CYP450) are three representative stress-responsive factors in response to various stresses. There were 8 HSP, 5 GST, and 12 CPY450 genes that were differentially expressed under low salinity stress (Supplementary Materials Figures S3–S5). Betaine acts as an osmoprotectant, maintaining osmotic balance and protecting cells from osmotic stress. Within the betaine synthesis pathway, the genes encoding hemocyanin and prophenoloxidase-2 (PPO2) were upregulated in shrimp under low salinity stress (Supplementary Materials Figure S6). *Na^+^/K^+^-ATPase* (NKA), *Na^+^/K^+^/2Cl^−^* cotransporter (NKCC), *V-type proton (H^+^)-ATPase* (VAT), *Carbonic anhydrase* (CA) are typical osmoregulation genes. Among them, five genes (three NKA and two NKCC) were differentially expressed upon salinity stress (Supplementary Materials Figure S7).

Taken together, *L. vannamei* can adapt to low salinity conditions by regulating free amino acids, lipids, stress-responsive factors, and osmoregulatory genes.

### 2.2. ATAC-Seq Analysis in Shrimp Under Low Salinity Stress

ATAC-seq analysis helps identify chromatin accessibility and cis-regulatory dynamics that underlie shrimp salinity adaptation. ATAC-seq was conducted under control (30‰) and low salinity (3‰) conditions. The obtained ATAC-seq reads densely distributed around gene body regions, with pronounced sequencing depth peaks noted around transcription start sites (TSS) and transcription end sites (TESs) (Supplementary Materials Figure S8). Approximately 300,000 peaks were identified per sample (*p* < 1× 10^−5^). By the cross-tabulation analysis of the biological replicates, we identified a set of 98,967 shared peaks within the control group and 112,604 peaks within the low salinity group. A peak distribution analysis showed that 15% of the peaks were within gene bodies, while 4% were near regulatory regions (potential promoter regions) (Supplementary Materials Figure S9).

Differential peak analysis was performed to compare chromatin accessibility between the control (30‰) and low salinity (3‰) groups. In total, 60,513 differential peaks (DPs) were identified, with 8874 (14.7%) in gene bodies and 7045 (11.6%) in promoter regions (3 kb upstream). A total of 5691 genes harbored DPs in their gene bodies or promoters, and some of these genes harbored one or more DPs (Supplementary Materials Table S1).

### 2.3. Integration of ATAC-Seq and RNA-Seq Reveals Unique Cis-Regulatory Mechanisms of Shrimp

**Figure 2:**
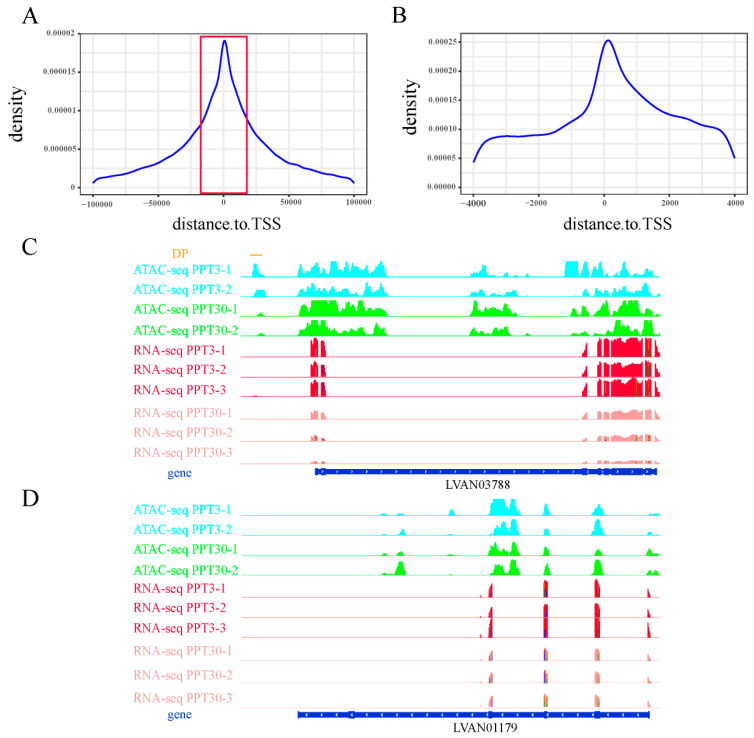
Distribution of DPs around transcription start sites (TSS): (**A**) Distribution of DPs around TSS. (**B**) A zoomed-in view of DP distribution of TSS (the red box of plot A). (**C**) The chromatin accessibility and expression profiles of a randomly selected DE-DPG. (**D**) The chromatin accessibility and expression profiles of a randomly selected DEG without any DPs.

The integration of ATAC-seq and RNA-seq helps identify which DEGs are regulated by TFs. Herein, genes hosting DPs (DPGs) within their promoter regions (3 kb upstream) are considered to be candidates regulated by TFs. A strong positive correlation was found between the DPs and the expression of nearby genes (correlation index = 0.38), confirming a significant connection between gene expression patterns and chromatin accessibility (Figure 1C). After excluding DPs with weak or negative correlations with gene expression, a total of 5449 DPs showed a significant positive correlation with the expression levels of the nearby genes. These DPs were predominantly concentrated near the transcription start site (TSS) (Figure 2A,B), suggesting that they might represent binding hotspots for TFs. Genes with DPs in their promoters showed differential expression profiles under salinity stress (Figure 2C), further supporting the role of DPs as TF binding sites regulating gene expression. Conversely, the absence of DPs near some DEGs (Figure 2D) implied that their differential expression might be regulated by alternative regulatory mechanisms beyond the scope of cis-regulation.

The integration of DEGs and DPGs initially identified 172 overlapping genes. To expand the analysis, we relaxed the DEG criteria (fold change > 2 and *p* < 0.5) and identified 637 DEGs near DPs (DE-DPGs) (Figure 1D). As expected, these DE-DPGs showed functional enrichments similar to DEGs, including the same enriched pathways of tyrosine metabolism, fat digestion and absorption, and betalain synthesis (Figure 1A,B). In addition, numerous stress-responsive genes, including three GST genes, three CYP450, and three HSP genes, were also included in these DE-DPGs.

Pathways such as the PI3K-AKT signaling pathway and steroid hormone biosynthesis were significantly enriched among DE-DPGs compared to DEGs, marking them as potentially direct targets of TF regulation (Figure 1B). The PI3K-AKT signaling pathway is well known for its crucial role in salinity acclimation. All the 15 DE-DPGs in the PI3K-AKT pathway were upregulated under low salinity stress (Supplementary Materials Figure S10), highlighting their pivotal role in TF-mediated salinity adaptation.

### 2.4. Identification of Salinity Adaptation-Related TFs in Shrimp

Sequence analysis of DPs provides important clues for identifying potential TFs with binding activity. Firstly, a genome-wide scan for TFs was carried out using TF-finder, resulting in identification of 790 genes encoding 68 distinct TFs within the shrimp genome. Using FIMO, 7,317,611 TFBSs and 885 TFs were predicted based on the 60,513 DP sequences (Supplementary Materials Figure S11). Then, an intersection analysis of these two sets (TFs predicted by TF-finder and FIMO) identified 13 common TFs. Among the 120 genes encoding these 13 TFs, 45 genes encoding nine TFs exhibited differential expression under low salinity stress. These nine TF genes were totally upregulated with decreasing salinity (Supplementary Materials Figure S12), indicating their active role in salinity adaptation. Therefore, these nine TFs are suggested to be the key cis-regulators of salinity acclimation in shrimp (Table 1).

### 2.5. Characteristics and Targeted Genes of the Nine TFs

The nine identified TFs include ZBTB, RFX, NRF1, ETS, TFCP2, MYB, ARID, bHLH, and NFAT5. Although NFAT5 and bHLH have been previously reported to be the key factors in salinity adaptation [15,16], the remaining seven TFs represent novel regulators of this process. The large number of promoter-associated TFBSs for bHLH (4008) underscores its pivotal role in salinity adaptation. ZBTB exhibited the largest number of promoter-associated TFBSs and targeted the most genes (434), implying its potential as a master regulator.

**Figure 3:**
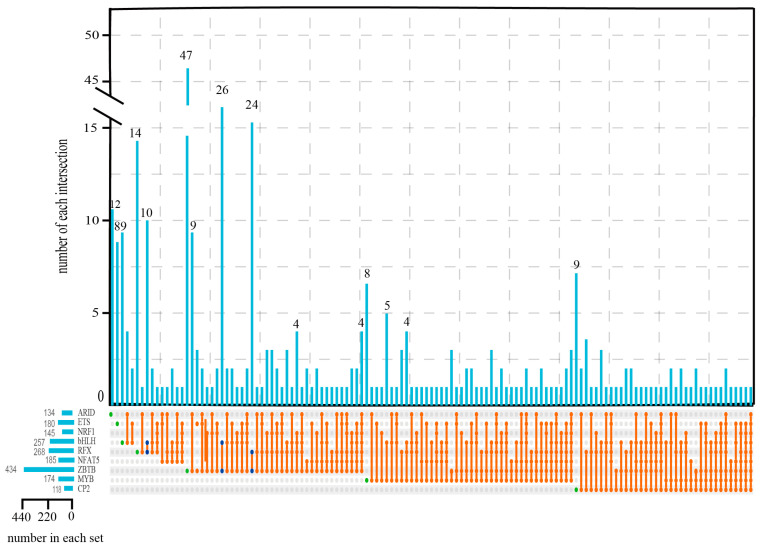
The intersection of the targeted DEGs of the nine TFs. The green circles represent the specific targeted DEGs of the correspondent TFs, and the blue circles represent the targeted DEGs shared between TFs.

We next characterized the TFBS motifs and their targeted DEGs for the nine identified TFs. By using JASPAR, we predicted the binding site motifs for each TF. Subsequently, the interactions between these motifs and their associated TFs were further confirmed through structure prediction using AlphaFold3. The nine TFs specifically targeted a large number of distinct DEGs (Figure 3, ZBTB: 47; ARID: 12; bHLH: 9; CP2: 9; ETS: 8; and MYB: 8). Moreover, significant overlaps in targeted DEGs were also observed among certain TF pairs (e.g., RFX-bHLH: 10, bHLH-ZBTB: 26, and RFX-ZBTB: 24) (Figure 3). This overlap suggested that RFX, bHLH, and ZBTB might co-regulate salinity adaptation through shared target genes.

#### 2.5.1. ZBTB, bHLH, and RFX

**Figure 4:**
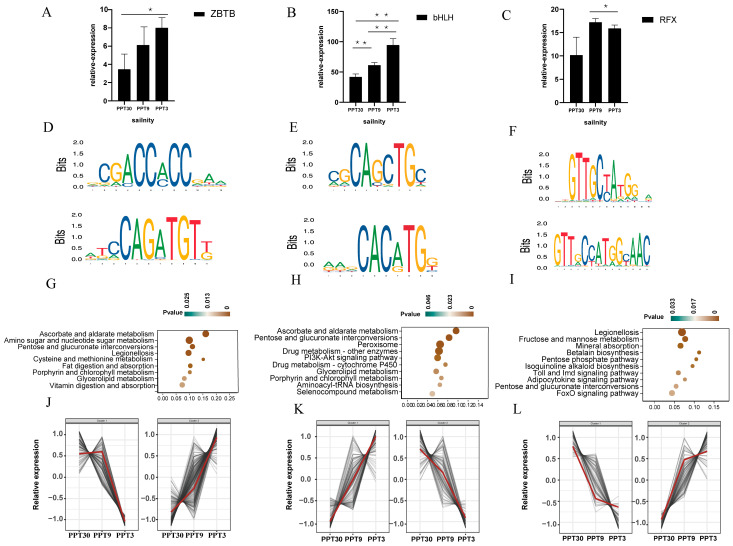
Basic characteristics of ZBTB, bHLH, and RFX: (**A**–**C**) Expression patterns (scale function) of ZBTB, bHLH, and RFX. * (*p* < 0.05) and ** (*p* < 0.01) indicate significant differential expression; (**D**–**F**) Conserved TFBS motifs of ZBTB, bHLH, and RFX (**G**–**I**) KEGG enrichment diagram of targeted genes of ZBTB, bHLH, and RFX; (**J**–**L**) Expression profiles of targeted genes of ZBTB, bHLH, and RFX in response to low salinity stress, the red line represents the overall expression trend of the targeted genes.

ZBTB, bHLH, and RFX are three TFs that share a large number of targeted genes. These three TFs were reported to regulate cell development, differentiation, environmental responses, and effector functions [17,18,19]. There were 22 ZBTB, 40 bHLH, and 5 RFX genes in the shrimp genome. Among them, 2 ZBTB, 23 bHLH, and 1 RFX genes were differentially expressed under salinity stress. As predicted by both JASPR and AlphaFold3, the conserved TFBS motifs (Supplementary Materials Figure S13A–C) of ZBTB were CAGATGT and CGACCXACC (Figure 4D). The TFBS motifs of bHLH were CAGCTGC and CACATG (Figure 4E), and for RFX they were GTTGCXAXG and GTTXCCATGGXAAC (Figure 4F). These three TFs targeted abundant DEGs (ZBTB: 434 DEGs, bHLH: 257 DEGs, and RFX: 268 DEGs) (Supplementary Materials Figure S14A–C), and these targeted DEGs were enriched in pathways of ascorbate/aldarate metabolism, pentose/glucuronate interconversions, and amino acid/lipid metabolism (Figure 4G–I). Notably, many osmoregulation genes (including NKA, NKCC, and V-ATPase) and stress-responsive genes (including HSP and CYP450) were also under the regulation of these three TFs. Collectively, ZBTB, bHLH, and RFX form a regulatory network that integrates metabolic and osmotic responses, enabling *L. vannamei* to adapt to low salinity stress.

#### 2.5.2. ETS, NFAT5, and MYB

**Figure 5:**
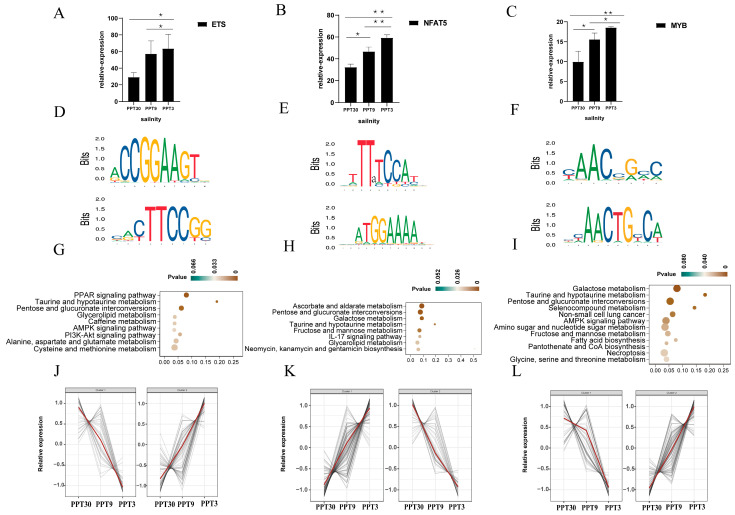
Basic characteristics of ETS, NFAT5, and MYB: (**A**–**C**) Expression patterns (scale function) of ETS, NFAT5, and MYB. * (*p* < 0.05) and ** (*p* < 0.01) indicate significant differential expression; (**D**–**F**) Conserved TFBS motifs of ETS, NFAT5, and MYB. (**G**–**I**) KEGG enrichment diagram of targeted genes of ETS, NFAT5, and MYB; (**J**–**L**) Expression profiles of targeted genes of ETS, NFAT5, and MYB in response to low salinity stress, the red line represents the overall expression trend of the targeted genes.

In comparison to the above three TFs, the other six TFs shared limited targeted genes. There were 21 ETS, 1 NFAT5, and 12 MYB genes in the shrimp genome. Among them, 11 ETS, 1 NFAT5, and 2 MYB genes were differentially expressed under salinity stress. The conserved TFBS motifs (Supplementary Materials Figure S13D–F) of ETS were CCGGAAGT and CTTCCGG (Figure 5D), for NFAT5 they were TTXTCCAT and ATGGAAAA (Figure 5E), and for MYB they were AACXGXC and AACTGXCA (Figure 5F). These three TFs targeted abundant DEGs (ETS: 180 DEGs, NFAT5: 185 DEGs, and MYB: 174 DEGs) (Supplementary Materials Figure S14D–F). The targeted genes of these three TFs were mainly enriched in the PI3K-AKT signaling pathway and some amino acid pathways (Figure 5G–I).

#### 2.5.3. NRF1, ARID, and CP2

**Figure 6:**
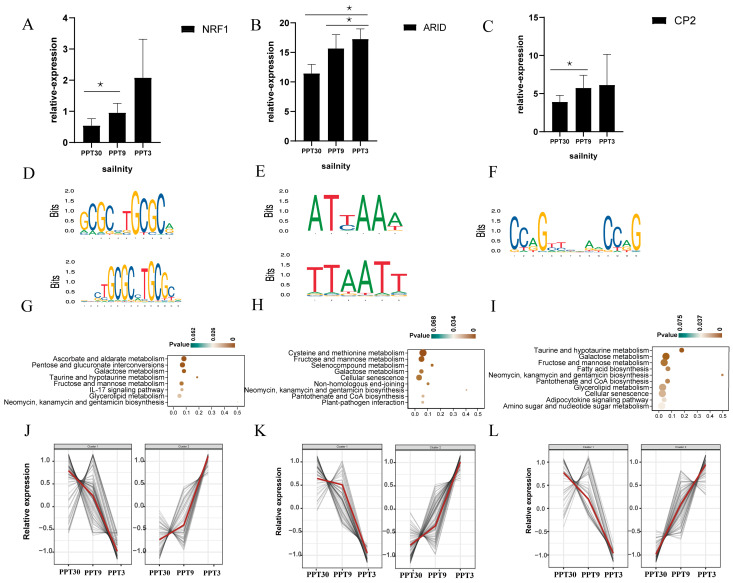
Basic characteristics of NRF1, ARID, and CP2: (**A**–**C**) Expression patterns (scale function) of NRF1, ARID, and CP2. * (*p* < 0.05) indicate significant differential expression; (**D**–**F**) Conserved TFBS motifs of NRF1, ARID, and CP2. (**G**–**I**) KEGG enrichment diagram of targeted genes of NRF1, ARID, and CP2; (**J**–**L**) Expression profiles of targeted genes of NRF1, ARID, and CP2 in response to low salinity stress, the red line represents the overall expression trend of the targeted genes.

NRF1, ARID, and CP2 are three TFs with few coding genes in the shrimp genome. There were only two NRF1, six ARID, and one CP2 genes in the shrimp genome. Among them, two NRF1, two ARID, and one CP2 genes were differentially expressed under salinity stress. The conserved TFBS motifs (Supplementary Materials Figure S13G–I) of NRF1 were GCGCXTGCGC (Figure 6D), for ARID were ATXAAA and TTAATT (Figure 6E), and for CP2 were CCAGXACCXG (Figure 6F). These three TFs targeted abundant DEGs (NRF1: 146 DEGs, ARID: 134 DEGs, and CP2: 118 DEGs) (Supplementary Materials Figure S14G–I). The targeted genes of these three TFs were mainly enriched in some amino acid and lipid metabolism pathways (Figure 6G–I).

## 3. Discussion

### 3.1. Cis-Regulation Mechanisms of Salinity Adaptation in Shrimp

Decapod crustaceans, such as *L. vannamei*, utilize well-established osmoregulation mechanisms to adapt to varying salinities, spanning from freshwater to saline environments [20]. However, the cis-regulation mechanisms and their associated elements and regulatory proteins (TFs) that underlie salinity adaptation remain elusive. In this study, we focused on the cis-regulation mechanisms of low salinity adaptation in *L. vannamei*. The hepatopancreas, an essential organ that participates in amino acid and lipid metabolism, energy homeostasis, and stress responses, was analyzed to elucidate this issue.

Chromatin openness not only directly impacts gene expression levels but also attracts TFs to regulate gene expression [21]. Gene expression levels can be influenced by a variety of factors, yet not all gene expression is regulated by TFs [22]. This interaction between TFs and functional genes forms the crux of the organism’s adaptive response to salinity changes [23,24]. Our findings revealed that only a subset of DEGs falls under the regulation by TFs. An in-depth study of the nuances of these DEGs provides valuable insights into the cis-regulatory mechanisms of salinity acclimation in shrimp. Thus, we performed an integration analysis of ATAC-seq and RNA-seq, and identified many salinity adaptation mechanisms under cis-regulation, including regulating amino acid and lipid metabolism, betalain synthesis and PI3K-AKT signaling pathways, and stress-responsive genes (GST, HSP, and CYP450).

Osmoregulation refers to the regulation of osmotic (and ionic) homeostasis within an organism relative to the ionic property of the surrounding environment. Similarly to other biological processes, osmoregulation is regulated by molecular events, in which the responsible genes can either work independently or in cooperation to effect the necessary changes [20]. Ion transportation and energy consumption are two fundamental processes in osmoregulation. Ion-motive osmoregulation-related enzymes, including NKA, CA, NKCC, and V-ATPase, are important in regulating the water and salt balance in crustaceans [20]. GST and CYP enzymes enhance ROS scavenging and promote homeostasis and defense mechanisms under salt stress in mouse liver and kidney tissue [25] by regulating Na^+^, K^+^, and Cl^−^ ion concentrations inside and outside the cell [26]. The significance of these enzymes is supported by their high expression levels in decapods in response to salinity stresses [20,27]. In this study, we found a large number of these osmoregulation-related enzymes were under the cis-regulation. Their chromatin accessibility and expression profiles were both significantly changed under low salinity stress.

Furthermore, numerous metabolic and signaling pathways associated with salinity adaptation were proposed to be subject to cis-regulatory control in *L. vannamei*. In many decapods, free amino acids, notably glycine, proline, and alanine, play important roles in modulating osmotic balance in response to the changes in salinity environments [28]. The concept of isosmotic intracellular regulation (IIR), primarily influenced by inorganic ions and free amino acids [20], underlines the complexity of osmoregulatory processes. In addition, the betalain synthesis and PI3K-AKT signaling pathways also play important roles in osmoregulation by positively regulating ion transportation in the gill tissue of the turbot fish and in the leaves of *Disphyma australe* [29,30]. Our results indicated that these metabolic and signaling pathways, especially for the free amino acid metabolism and PI3K-AKT signaling pathways, were governed by cis-regulatory mechanisms.

### 3.2. The Hepatopancreas: A Central Hub for Metabolic and Osmotic Adaptation to Low Salinity Stress

The hepatopancreas, as a multifunctional organ, plays a pivotal role in low salinity adaptation by integrating metabolic and osmotic responses in *L. vannamei* [31]. Unlike gill tissue, which primarily regulates ion transport, the hepatopancreas is responsible for amino acid and lipid metabolism, energy production, and detoxification processes in Scylla paramamosain [32]. Under low salinity stress, the hepatopancreas upregulates pathways such as amino acid metabolism (e.g., glycine, serine, and threonine metabolism) and lipid metabolism (e.g., fat digestion and absorption), which are essential for maintaining osmotic balance and energy homeostasis in crustaceans [33]. Additionally, the hepatopancreas modulates stress responses through the upregulation of genes encoding heat shock proteins (HSPs) and cytochrome P450 enzymes (CYP450), which protect cells from oxidative damage and enhance detoxification capacity [34]. These findings highlight the hepatopancreas’s unique role in systemic adaptation to low salinity stress, complementing the osmoregulatory functions of gill tissue.

### 3.3. Novel Osmoregulation-Related TFs Identified in Shrimp

NFAT5 and bHLH, which are known for their roles in osmoregulation in gill tissue [15] are also active in the hepatopancreas, suggesting their systemic role in salinity adaptation. For example, NFAT5 regulates betaine transporters and osmolyte synthesis in response to osmotic stress; meanwhile, bHLH modulates antioxidant responses and iron homeostasis, both of which are crucial for the function of the hepatopancreas under low salinity conditions. This study successfully identified nine TFs within the *L. vannamei* genome supposed to significantly impact salinity adaptation capabilities. Among them, NFAT5 and bHLH are well known for their regulatory roles in salinity adaptation. Specifically, NFAT5 can be activated and translocated to the nucleus under salinity stress, subsequently activating the expression of certain osmoregulation-related genes and betaine transporter genes [24]. bHLH has been more extensively studied in plants, which regulates abscisic acid metabolism [35], osmoregulation, and iron acquisition [36]. In line with these reports, the targeted genes of NFAT5 and bHLH were found to be significantly enriched in several key metabolic and signaling pathways, including ascorbate and aldarate metabolism, pentose and glucuronate interconversions, cytochrome P450 metabolism, the PI3K-Akt signaling, taurine and hypotaurine metabolism, and steroid hormone biosynthesis. These pathways, as documented in previous research, are intimately connected with osmoregulatory processes [20,37,38,39,40]. These results suggested that penaeid shrimp may indeed deploy analogous cis-regulatory mechanisms for salinity adaptation similar to those observed across different biological systems.

In addition to NFAT5 and bHLH, we identified seven novel TFs potentially involved in salinity adaptation. For example, ZBTB and RFX, which were previously associated with cell differentiation and development in other species [17,19], may play unique roles in the hepatopancreas under low salinity stress. ZBTB, with its extensive target gene network, may regulate lipid metabolism and energy homeostasis, in contrast, RFX could modulate amino acid metabolism and stress responses, highlighting the hepatopancreas’s central role in metabolic adaptation. Considering the impressive osmotolerance of penaeid shrimp, we proposed the hypothesis that novel TFs, beyond the conventional suspects, could be actively engaged in facilitating salinity adaptation. As shown in previous studies, these seven TFs are majorly involved in controlling cell differentiation, proliferation, and survival [17,41,42]; regulating development; maintaining cellular homeostasis [43]; regulating various material and energy metabolisms; and the basic life activities of organisms [44]. Although their direct impact on osmoregulation requires further elucidation, their involvement in processes that intersect with osmoregulatory pathways hints at a potentially broader role in this context. Indeed, the targeted genes of these TFs were found to intersect significantly with key osmoregulation-related genes and pathways, including the amino acid and lipid metabolism pathways, betalain synthesis pathway, PI3K-Akt signaling pathway, and osmoregulatory and stress-responsive genes. Notably, ZBTB and RFX exhibited a considerable overlap in their targeted gene repertoire with the well-characterized osmoregulation of TFs NFAT5 and bHLH, suggesting that they may converge on similar regulatory mechanisms in the osmoregulation process.

Besides similarities, these TFs may each execute distinct, specialized roles in the intricate process of salinity adaptation. Abundant TF-specific targeted genes were identified in these TFs, particularly evident in the cases of ZBTB, RFX, and ARID. Despite the current gaps in the understanding of the precise functions and regulation mechanisms of these TFs, it can be reasonably inferred that they likely undertake unique responsibilities in shrimp for salinity adaptation. As inhabitants of environments ranging from brackish waters (with a mere 1‰ salinity) to highly saline waters (reaching up to 40‰ salinity) [2,3], penaeid shrimp possibly employ unique osmoregulation strategies and TF-mediated regulations. The novel association of these TFs with osmoregulation provides valuable resources for the delineation of specific adaptive mechanisms employed by shrimp in response to salinity stresses. These TFs may form a complex cis-regulatory network in the hepatopancreas, enabling shrimp to adapt to varying salinities. For instance, ZBTB and RFX share targeted genes with NFAT5 and bHLH, suggesting overlapping regulatory mechanisms, while also targeting unique genes involved in amino acid and lipid metabolism, which are critical for hepatopancreas function under low salinity stress. This network likely integrates metabolic and osmotic responses, ensuring systemic adaptation to environmental changes.

## 4. Materials and Methods

### 4.1. Shrimp Sampling for ATAC-Seq and RNA-Seq

For ATAC-seq and RNA-seq, the same samples of *L. vannamei* under low salinity stress were collected. A total of 20 *L. vannamei* were stocked and cultured in each experimental group under different salinity conditions, and a total of 180 *L. vannamei* shrimps were used. Subsequently, three *L. vannamei* were randomly sampled from each group for sequencing (Supplementary Materials Tables S2 and S3). Randomly selected adult shrimp of *L. vannamei* that are healthy and free from pathogenic bacteria infection (7.2 ± 0.5 cm) were cultured and acclimated to a salinity of 30‰ for two weeks at the shrimp culture laboratory of the Institute of Oceanology Chinese Academy of Sciences in Qingdao, China. The salinity was gradually reduced from 30‰ to 3‰ at 3‰ per day when reducing salinity from 30‰ to 9‰, and 1‰ per day when reducing salinity from 9‰ to 3‰. Bait was fed three times a day, water was changed once a day, and water temperature was controlled at 25 °C. The animals were allowed to acclimate to salinities of 9‰ and 3‰ for 24 h. Then, randomly selected hepatopancreas samples were collected from the animals and acclimated to salinities of 30‰, 9‰, and 3‰, which were used for the subsequent ATAC-seq and RNA-seq.

### 4.2. RNA-Seq and Analyses

Hepatopancreas samples under salinities of 30‰ (the Control group), 9‰, and 3‰ were used for RNA-seq with three replicates. Two experimental salinity gradients (9‰ and 3‰) were established to investigate the trends in expression profiles under decreasing salinity conditions. According to the standard manufacturer’s protocol, total RNA was isolated and purified from the samples using a TRIzol extraction reagent (Thermo Fisher Scientific, Waltham, MA, USA). RNA quality was determined by 1% agarose gel electrophoresis, and RNA concentration was assessed using a Nanodrop 2000 spectrophotometer (Thermo Fisher Scientific, USA). Transcriptome libraries were prepared according to the instructions of the TruSeq RNA Library Prep Kit (Illumina, San Diego, CA, USA), and then sequenced on the Illumina HiSeq 2500 platform. The TopHat v1.2.1 package was used to map the transcriptome reads to the shrimp genomes (GCA_003789085.1) [45]. Then, fragments per kilobase of transcript per million fragments mapped (FPKM) were calculated using Cufflinks v2.2.1 (http://cole-trapnell-lab.github.io/cufflinks/) (accessed on 3 May 2024). The differential gene expression analysis was conducted by using edgeR V3.10 [46]. The intragroup dispersion was computed when we first calculated the mean value of the measured variable for each group. Then, for each data point in the group, we subtracted the group mean, squared the result, and summed these squared differences. This sum was divided by the number of data points minus one, and the square root of the quotient was taken as the standard deviation.

### 4.3. ATAC-Seq and Analyses

Hepatopancreas samples under salinities of 30‰ and 3‰ were used for ATAC-seq with two replicates. The samples were spheroplasted prior to incubating with Nextera Tn5 Transposase (Illumina, San Diego, CA, USA). After the transposition reaction, DNA purification, and PCR amplification, the libraries were prepared according to the protocols of ATAC-seq [47]. The ATAC-seq and Nextera workflow were designed for sequencing according to Illumina high-throughput sequencing instruments (Illumina, San Diego, CA, USA). The sequencing reads were mapped to the shrimp genome (GCA_003789085.1) [45] using Bowtie2 [48]. Peak calling was performed using MACS v2.1.0 with a *p*-value cutoff of 1 × 10^−5^ and the broad flag, and then filtered based on mappability [49]. Common peaks in replicate samples were called and used for the following comparative analyses: DESeq2 was used for differential peak identification with a nominal *p*-value cutoff of 1 × 10^−5^. The peaks detected in the 3 kb upstream region of TSS were defined as the proximal peaks, and those detected in the upstream 3 kb to 100 kb region of TSS were defined as the distal peaks. All the differential proximal peak-associated genes (DPGs) were retrieved.

### 4.4. Integrative Analysis of ATAC-Seq and RNA-Seq

Genomic data visualization, including gene structure annotation, RNA-seq, and ATAC-seq profiles, was performed using Integrative Genomics Viewer (IGV) version 2.17.4 [50]. To investigate the correlation between the level of chromatin accessibility and gene expression, a correlation analysis was performed on the fold changes in the normalized read density values of DPs and expression levels of DPGs. The intersection of DPGs and DEGs (DE-DPGs) was calculated and regarded as the potential targeted genes of TFs. The functional annotation and pathway enrichment analysis of DE-DPGs were performed using the Kyoto Encyclopedia of Genes and Genomes (KEGG) database [51] and gene expression data were normalized using the scale function. The mean and standard deviation were calculated for each column of the gene expression data matrix, and then each data point was standardized by subtracting the column mean and dividing by the standard deviation.

### 4.5. TF and TFBS Prediction

Transcription factor binding sites (TFBSs) were predicted using FIMO (Find Individual Motif Occurrences) from the MEME Suite website (https://meme-suite.org/meme/) (accessed on 15 May 2024) with a significance threshold of *p*-value < 1 × 10^−4^ based on the DPs of *L. vannamei*. Position frequency matrices (PFMs) from the JASPAR CORE database for insect species were utilized for motif prediction. Furthermore, a genome-wide identification of TFs was performed using the TF-finder software (https://github.com/cjgunase/TF-finder) (accessed on 16 May 2024) [52]. The consensus transcription factors identified by both FIMO and TF-finder were determined through intersection analysis. The potential targeted genes of TFs were identified according to the locations of TFBSs around each gene. The genes located within a 3 kb window upstream or downstream of predicted TFBSs were considered to be potential target genes.

### 4.6. TFBS Prediction by AlphaFold3

To validate the relationship between TFs and their targeted genes, we predicted the interactions of promoters and TFs by AlphaFold3. Promoter regions harboring predicted TFBSs, as identified by FIMO analysis, were selected for structural modeling. The DNA sequences of promoter regions and the corresponding TF protein sequences were used as input for protein-DNA interaction prediction using AlphaFold3 (https://golgi.sandbox.google.com/) (accessed on 25 May 2024) [53]. The reverse complement of the promoter was also used for the AlphaFold3 prediction to ensure the promoter regions are represented as double-stranded structures. The confidential interaction models were visualized and analyzed using the PyMOL molecular graphics system (version 2.5.0).

## 5. Conclusions

In this study, we integrated ATAC-seq and RNA-seq analyses to investigate the molecular mechanisms of salinity adaptation in *L. vannamei*. Our results revealed a positive correlation between chromatin accessibility and gene expression under low salinity stress. Many DEGs with differential chromatin accessibility (DE-DPGs) were suggested to be under cis-regulation in response to the low salinity stress. These DE-DPGs were majorly involved in osmoregulation-related genes (including NKA, CA, NKCC, V-ATPase, HSP, GST, and CYP450) and pathways (including the free amino acid and lipid metabolism, betalain synthesis, and PI3K-ART signaling pathways). Based on the results of the integrated analyses, two known and seven previously unrecognized osmoregulation-related TFs were identified. The characterizations of the nine TFs were thoroughly investigated, including their functions, expression patterns, and the regulatory landscape of their target genes. This study provides new insights into the unique cis-regulatory mechanisms that enable *L. vannamei* to adapt to extremely low salinity conditions and offer novel osmoregulation-related TFs for further research on shrimp aquaculture and environmental adaptation.

## Figures and Tables

**Table 1:** Basic characteristics of the nine salinity adaptation-related TFs.

TF	Full Name	Encoding Gene Number (DEGs)	Score *	*p*-Value *	Expression Profile	TFBS Number	TFBSs in Promoter	Targeted Gene Number
ZBTB	Zinc finger and BTB domain	2	21.2273	2.99 × 10^−9^	up	63,247	6064	434
CP2	Transcription factor CP2	1	18.202	3.14 × 10^−8^	up	11,537	1561	118
MYB	Myeloblastosis	2	22.3279	6.21 × 10^−9^	up	15,068	2817	174
NFAT5	Nuclear factor of activated T-cells 5	1	16.0845	1.85 × 10^−7^	up	16,368	2814	185
RFX	Regulatory Factor X	1	25.4138	7.25 × 10^−11^	up	54,216	3725	268
bHLH	Basic helix-loop-helix	23	17.4545	1.02 × 10^−7^	up	59,169	4008	257
NRF1	Nuclear factor E2-related factor 1	2	22.4634	2.24 × 10^−9^	up	26,183	2700	145
ETS	Erythroblast transformation-specific	11	20.1897	1.82 × 10^−9^	up	18,086	3025	180
ARID	AT-rich interactive domain	2	15.6071	2.40 × 10^−8^	up	11,149	2331	134

* score represents the motif’s affinity to bind at a specific sequence location, calculated via a position-dependent scoring matrix (PSSM). The *p*-value is calculated to assess the significance of motif occurrence.

## Data Availability

The RNA-seq and ATAC-seq data of *L. vannamei* were deposited in NCBI GenBank with the BioProject accession number PRJNA1036024.

## Supplementary Materials

The following supporting information can be downloaded at https://www.mdpi.com/article/10.3390/ijms26104605/s1.
