# Supplementary material for: Integrative ATAC-Seq and RNA-Seq Analysis Reveals Key Transcription Factors Mediating Low Salinity Adaptation in Penaeid Shrimp
Source: Int J Mol Sci. 2025 May 11;26(10):4605. doi: 10.3390/ijms26104605 (PMC12111515; doi:10.3390/ijms26104605)
Supplement: Supplementary file 1 [file ijms-26-04605-s001.zip › ijms-3570244-supplementary.pdf]

Supplementary materials for:

Integrative ATAC-seq and RNA-seq analysis reveals key transcription factors mediating low salinity adaptation in penaeid shrimp

Chuntao Zhang[1,3], Jianbo Yuan[1,2,4*], Roujing Li[1,3], Zhanyuan Yang[1,2], Man Luo[1,3], Xiaoyun Zhong[1,3], Jie Hu[1,3], Shuqing Si[1,3], Xiaojun Zhang[1,2,4], Fuhua Li[1,2,4]

[1] CAS and Shandong Province Key Laboratory of Experimental Marine Biology, Center for Ocean Mega-Science, Institute of Oceanology, Chinese Academy of Sciences, Qingdao, China.

[2] Key Laboratory of Breeding Biotechnology and Sustainable Aquaculture, Chinese Academy of Sciences, Wuhan, China.

[3] University of Chinese Academy of Sciences, Beijing, China

[4] Laboratory for Marine Biology and Biotechnology, Qingdao Marine Science and Technology Center, Qingdao, China.

* Correspondence and requests for materials should be addressed to J.Y. (yuanjb@qdio.ac.cn, Orcid ID: 0000-0002-8905-7438).

Figures

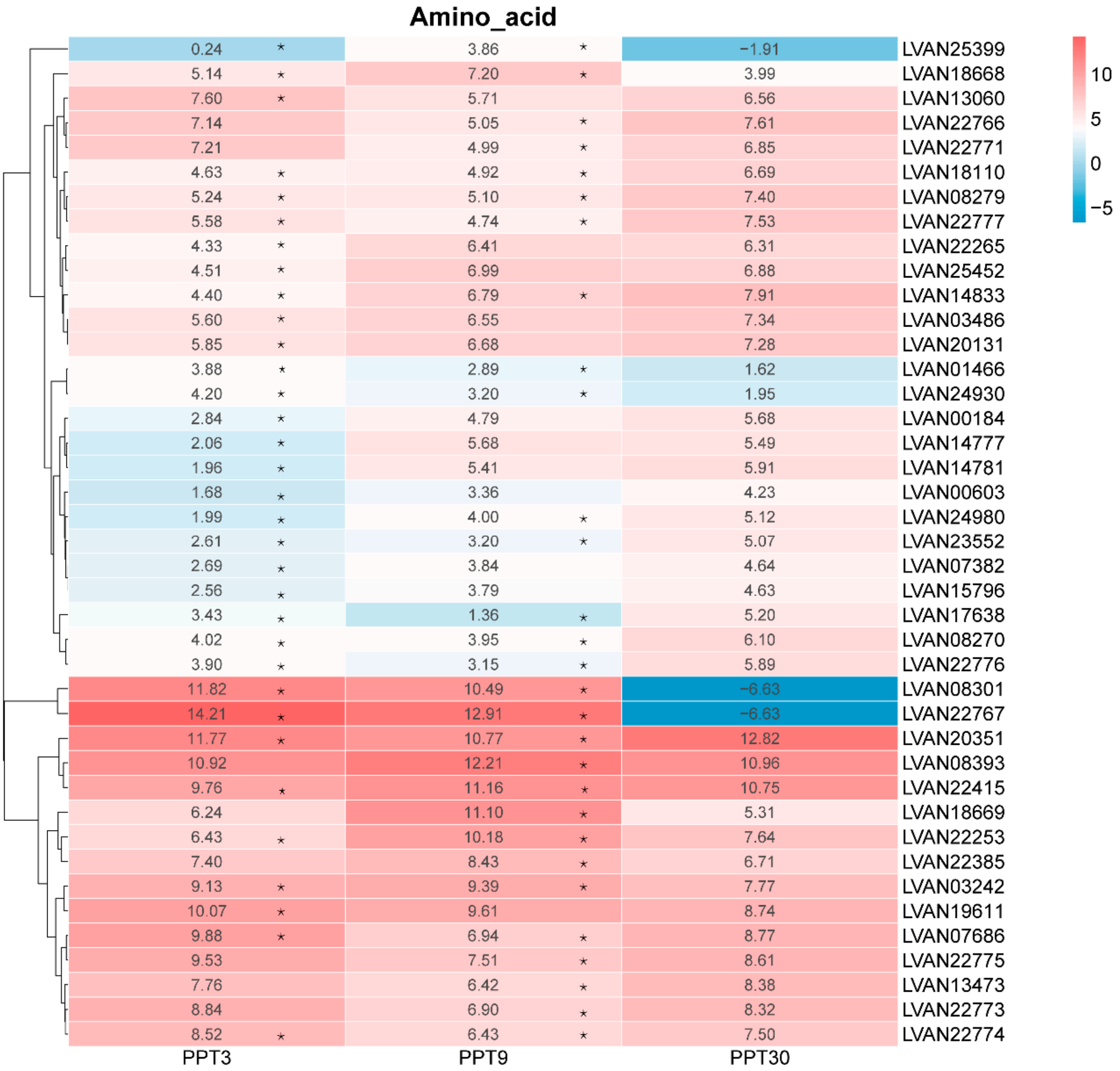

Fig. S1 Heatmap showing DEGs of free amino acid metabolic pathways. "*" indicates significant differential expression ($p < 0.05$) of the *L. vannamei* among the salinities of 30‰ (control group), 9‰, and 3‰.

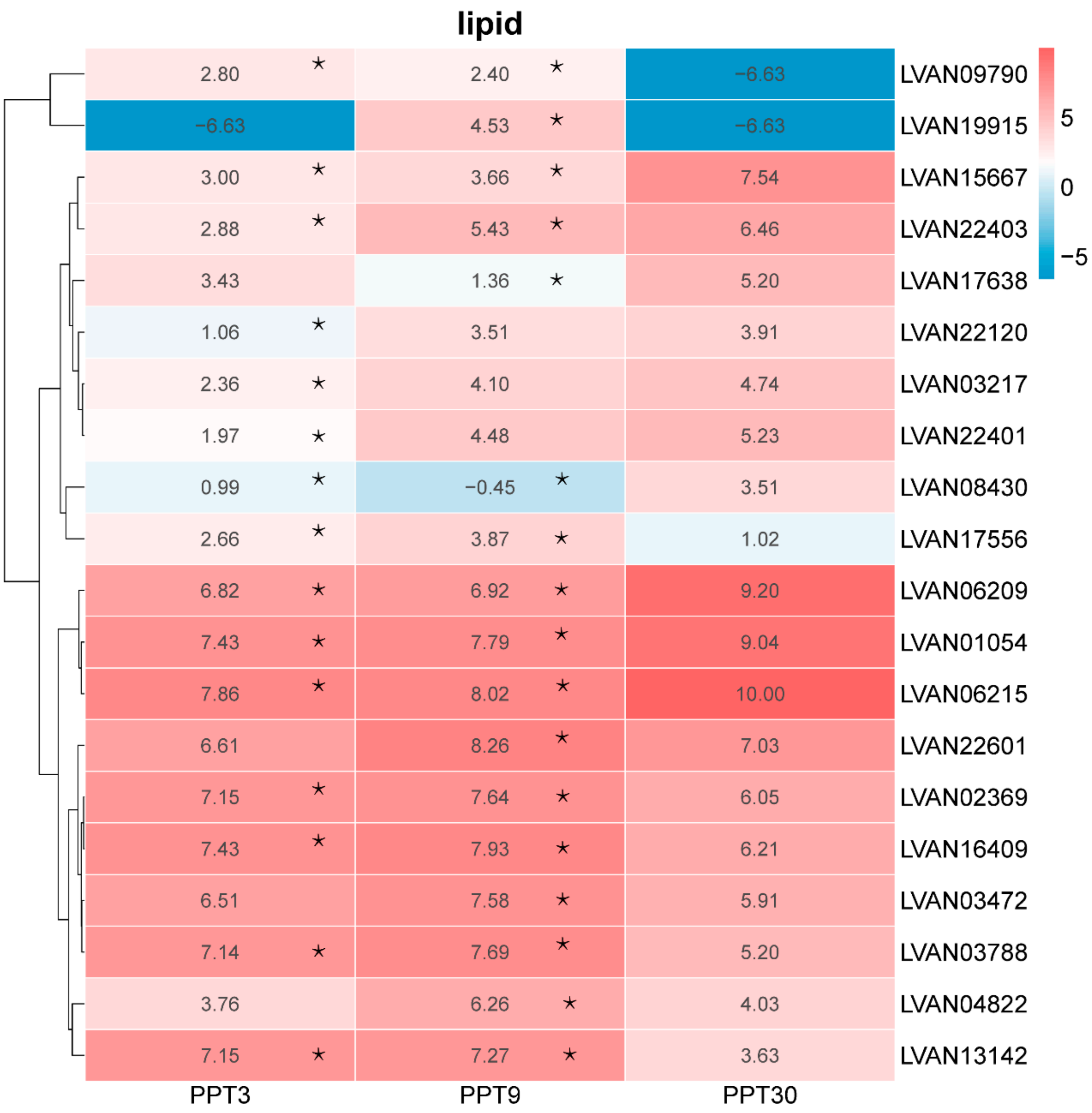

Fig. S2 Heatmap showing DEGs of lipid metabolic pathways. “*” indicates significant differential expression ($p < 0.05$) of the *L. vannamei* among the salinities of 30‰ (control group), 9‰, and 3‰.

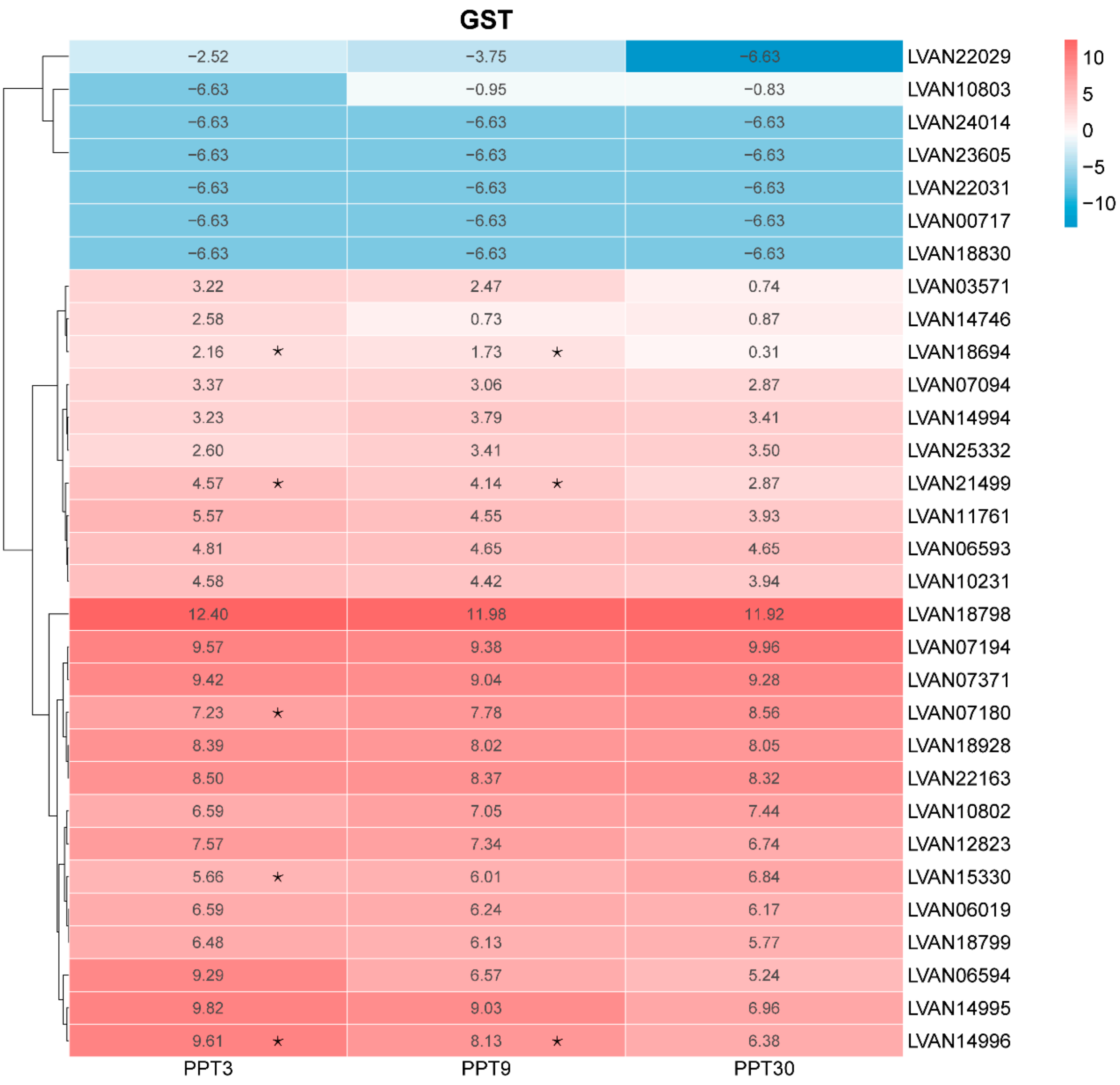

Fig. S3 Heatmap showing DEGs of stress factors Glutathione S-Transferases (GST). "*" indicates significant differential expression ($p < 0.05$) of the *L. vannamei* among the salinities of 30‰ (control group), 9‰, and 3‰.

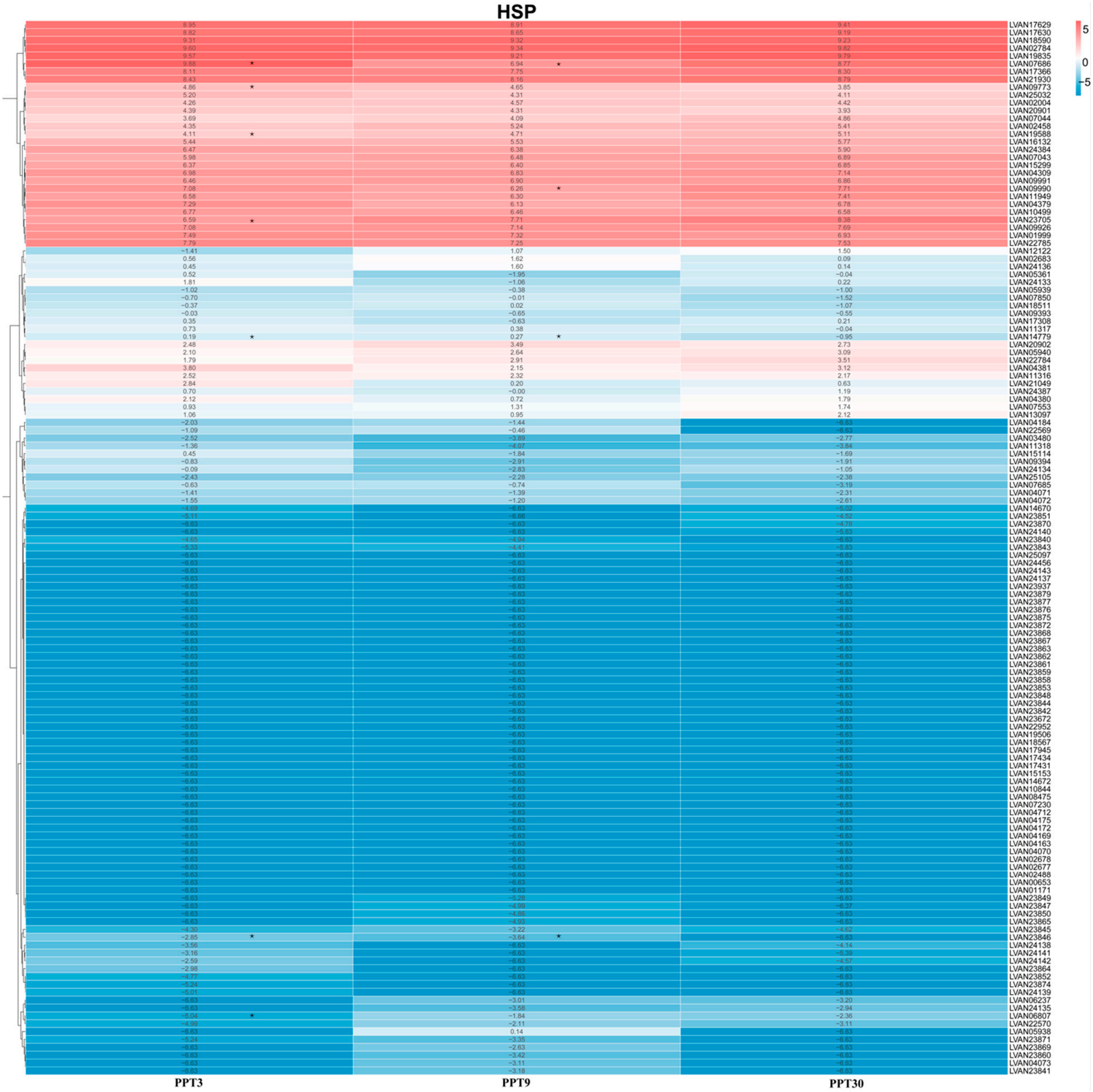

Fig. S4 Heatmap showing DEGs of stress factors heat shock proteins (HSP). "*" indicates significant differential expression ($p < 0.05$) of the *L. vannamei* among the salinities of 30‰ (control group), 9‰, and 3‰.

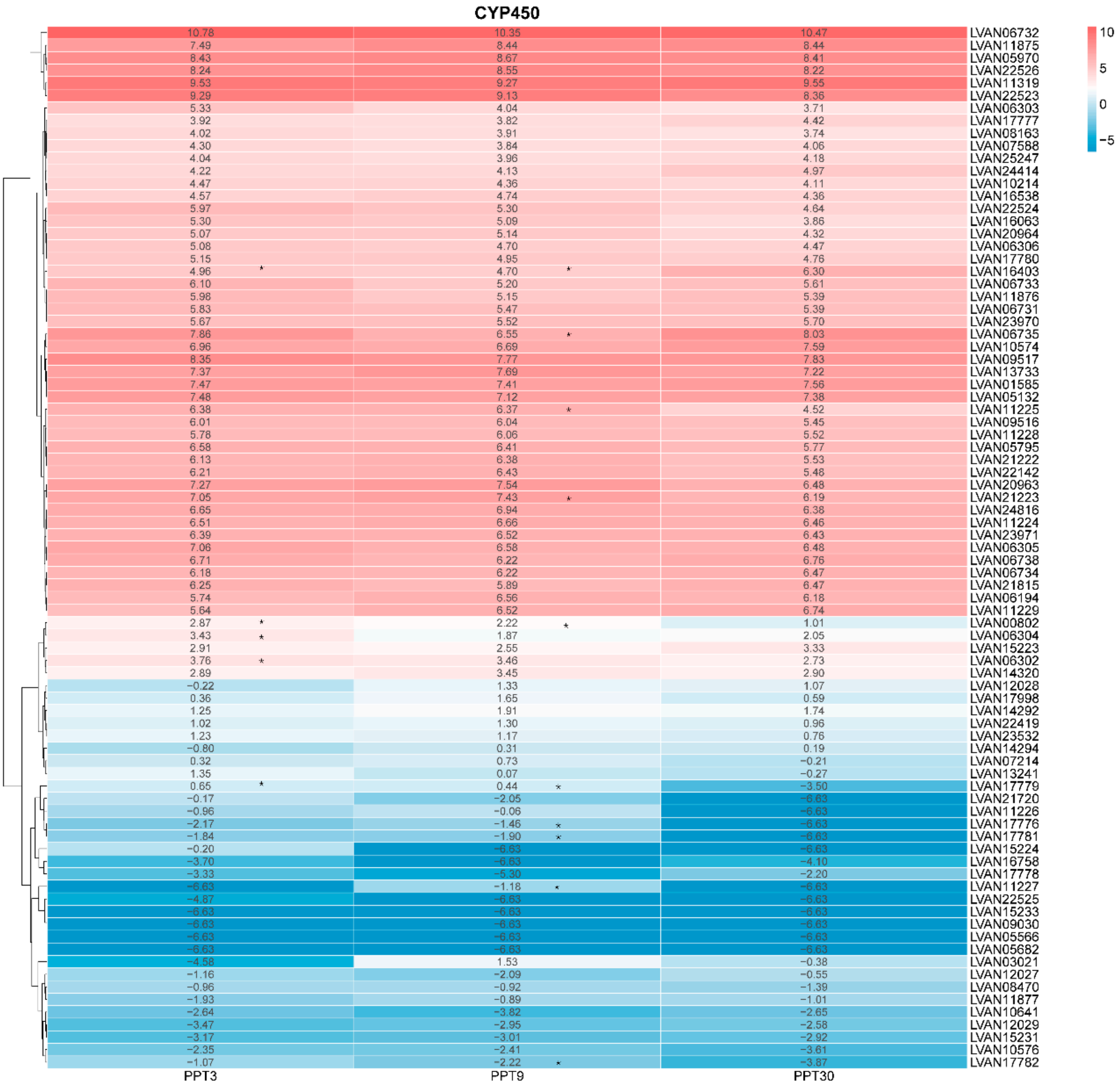

Fig. S5 Heatmap showing DEGs of cytochrome P450 (CYP450). “*” indicates significant differential expression ($p < 0.05$) of the *L. vannamei* among the salinities of 30‰ (control group), 9‰, and 3‰.

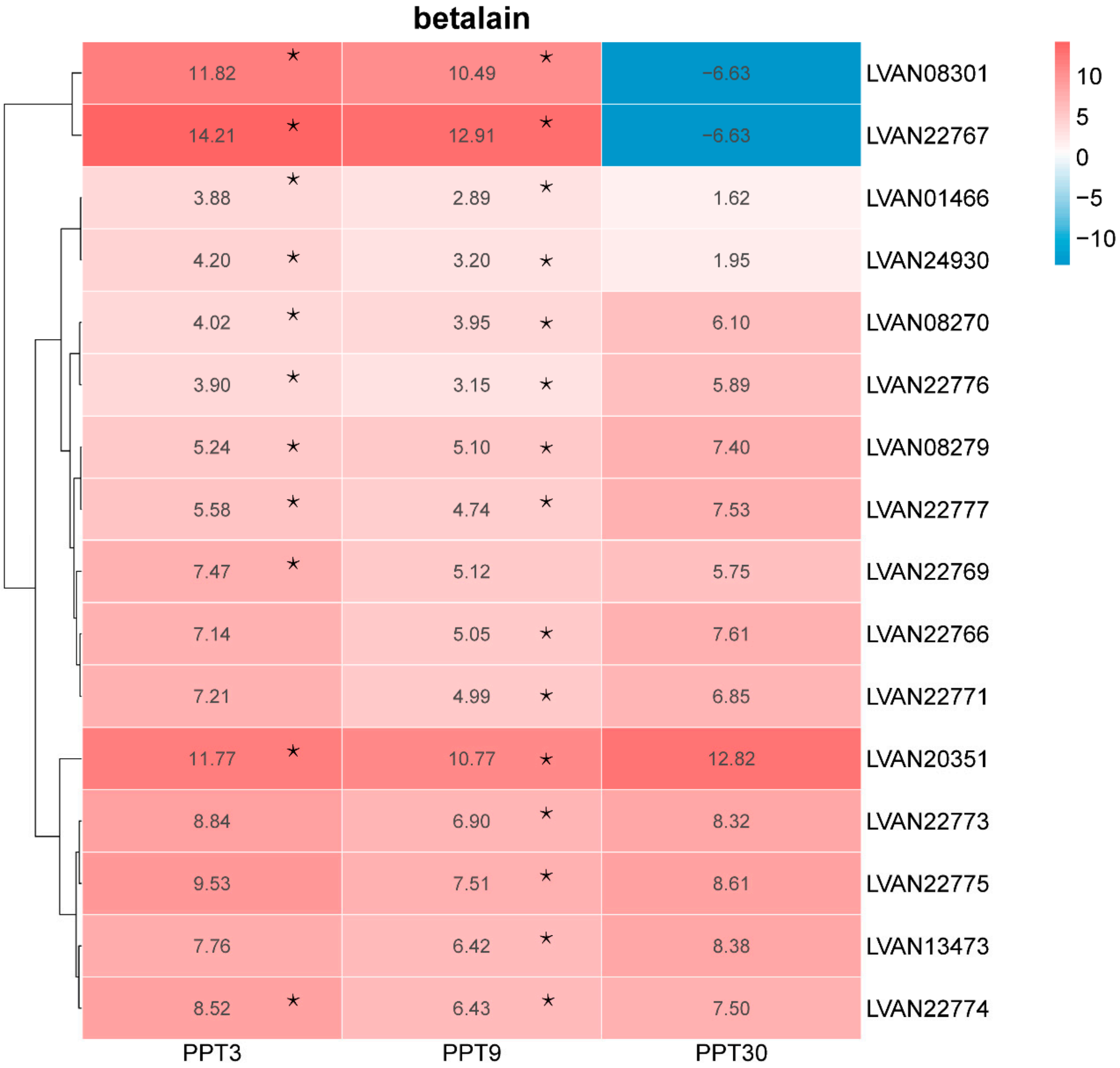

Fig. S6 Heatmap showing DEGs of hemocyanin and prophenoloxidase-2 (PPO2) in the betaine synthesis pathway. "*" indicates significant differential expression ($p < 0.05$) of the *L. vannamei* among the salinities of 30‰ (control group), 9‰, and 3‰.

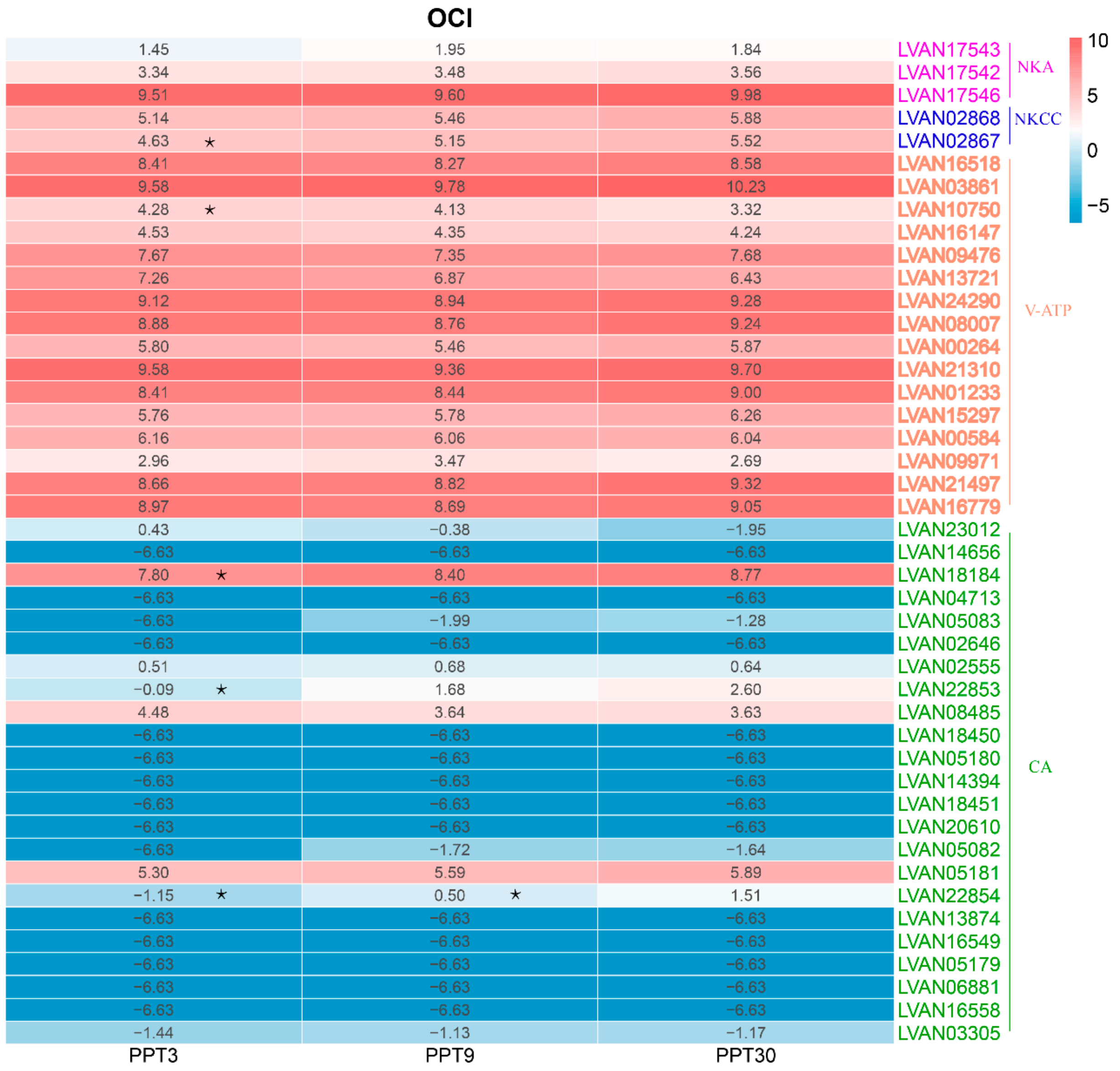

Fig. S7 Heatmap showing differential expression of osmoregulation genes, "*" indicates significant differential expression ($p < 0.05$) of the *L. vannamei* among the salinities of 30‰ (control group), 9‰, and 3‰. It contains NKA (Na+/K+-ATPase), NKCC (Na+/K+/2Cl− cotransporter), V-ATPase (Vacuolar H+-ATPase), CA (Carbonic anhydrase).

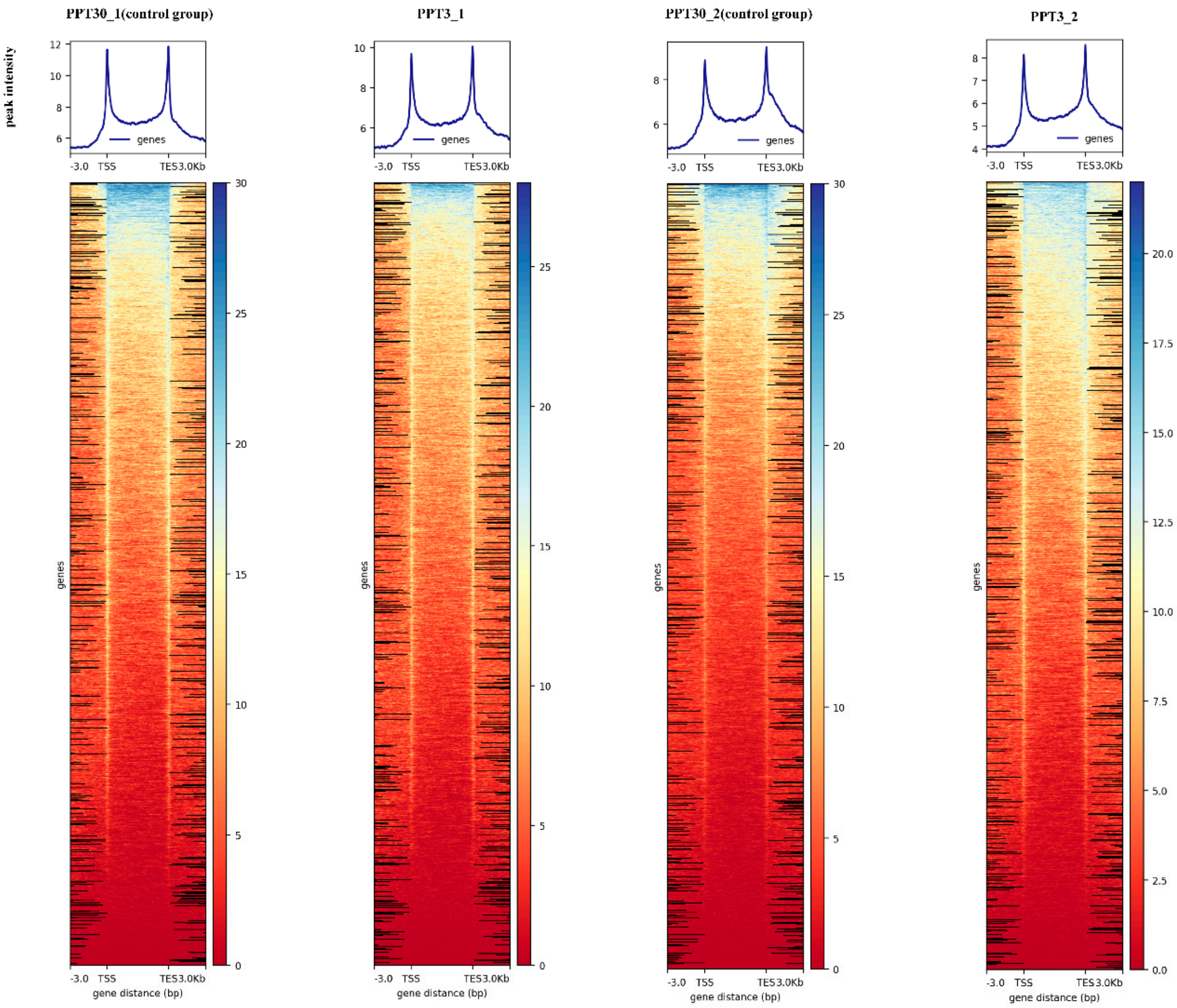

Fig. S8 The plots and heatmaps of ATAC-seq signals of over-enriched peaks. TSS indicates the transcription start sites. TES indicates the transcription end sites. PPT30 indicates the salinities of 30‰ (control group). PPT3 indicates the low-salinity stress condition (3‰), a total of two biological replicates. Peaks in the heatmaps are ranked from highest ATAC-seq signal (top) to lowest (bottom).

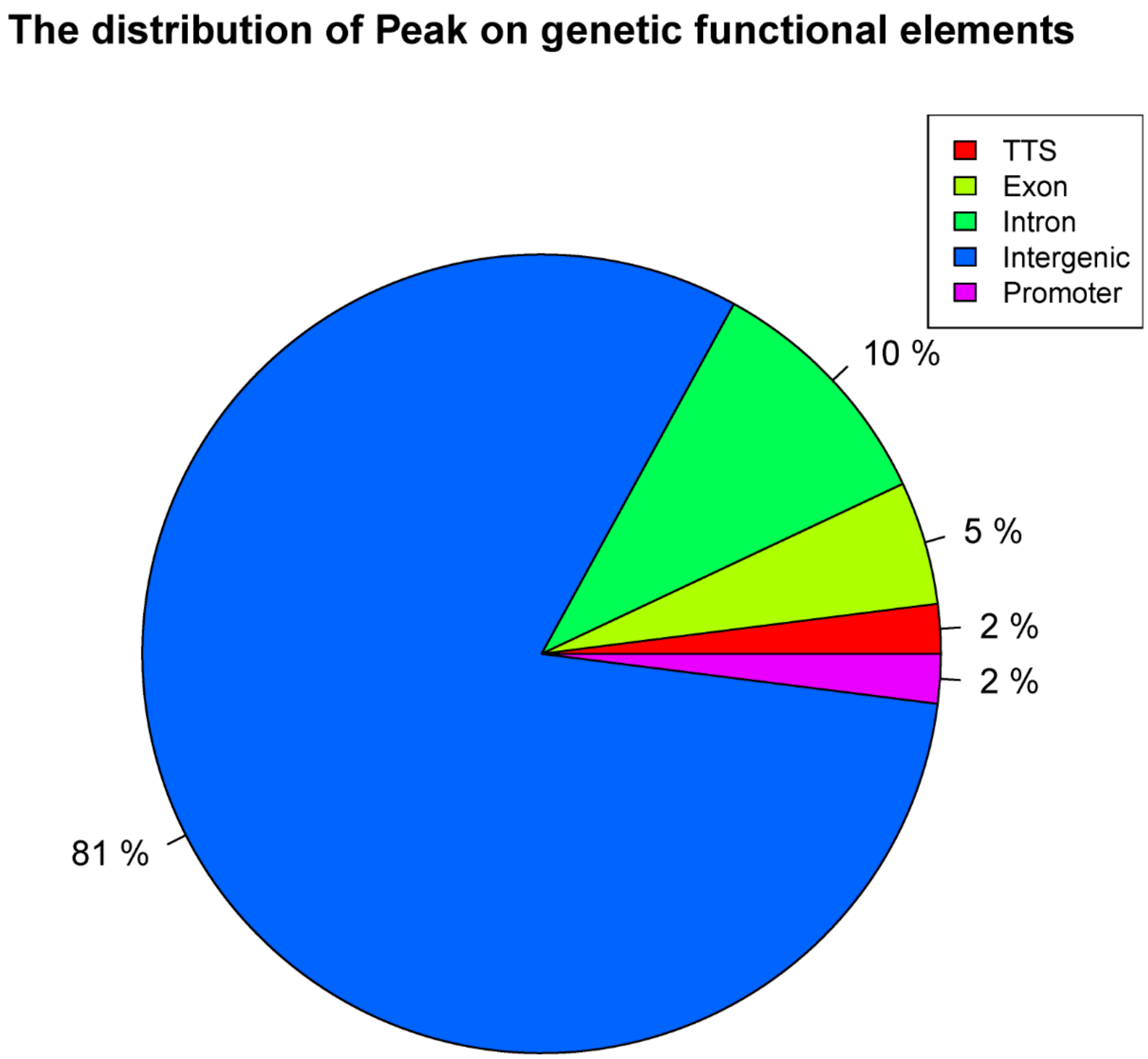

Fig. S9 Venn diagram showing the distribution of peak on genetic functional elements.

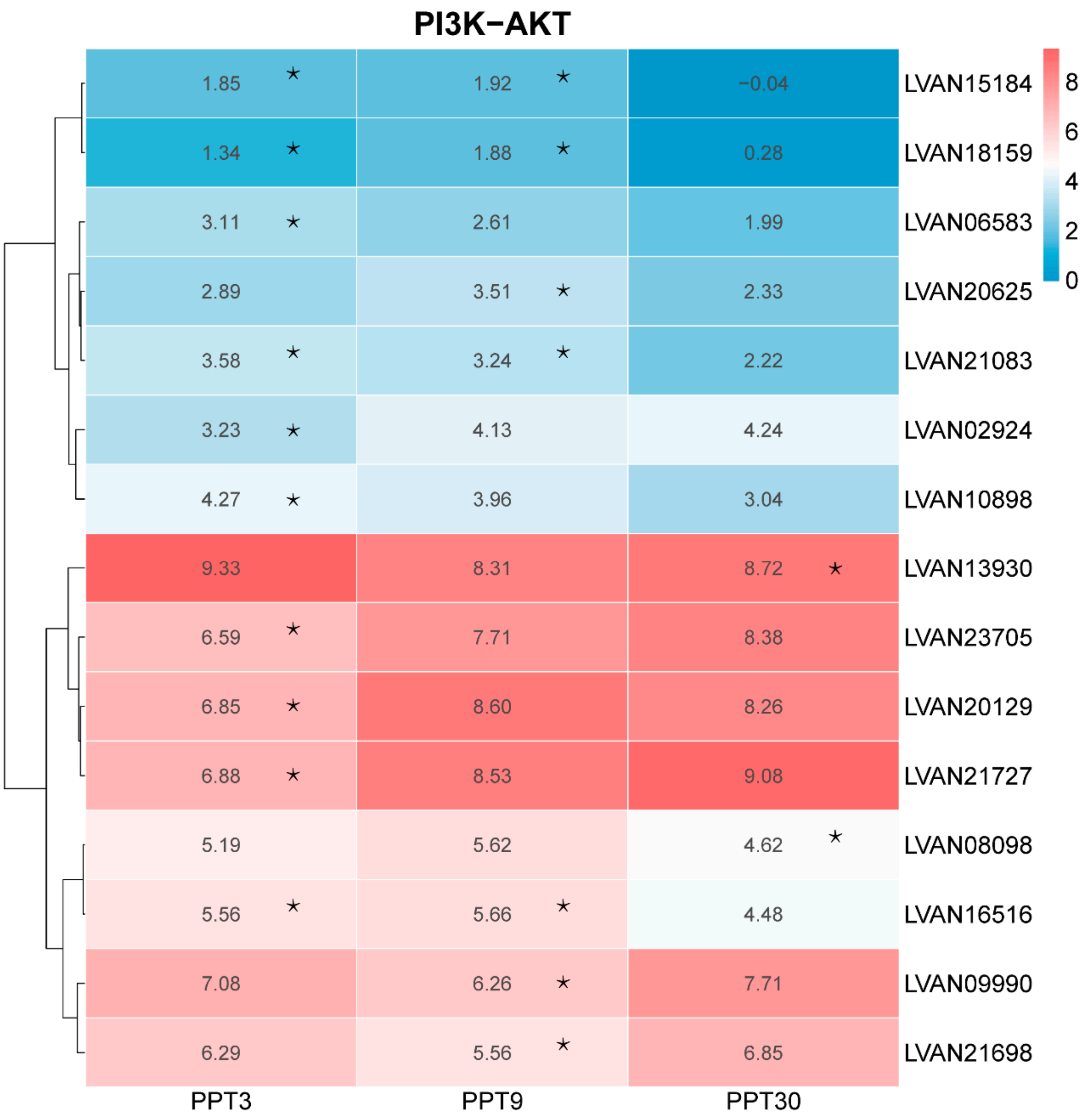

Fig. S10 Heatmap showing DP-DEGs of PI3K-Akt signaling pathway. "*" indicates significant differential expression (p < 0.05) of the *L. vannamei* among the salinities of 30‰ (control group), 9‰, and 3‰.

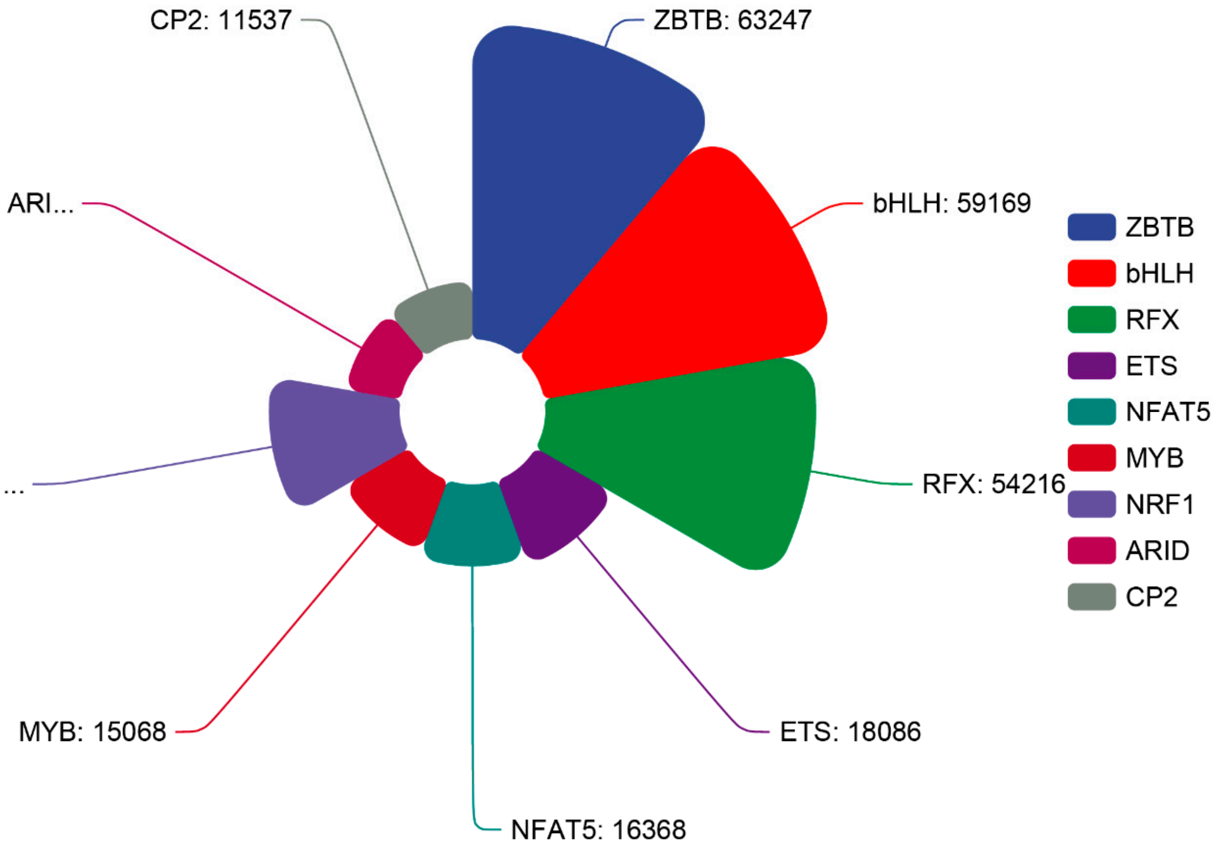

Fig. S11 Nightingale rose diagram showing the number of transcription factor binding sites (TFBSs) for nine transcription factors (TFs) associated with salinity adaptation

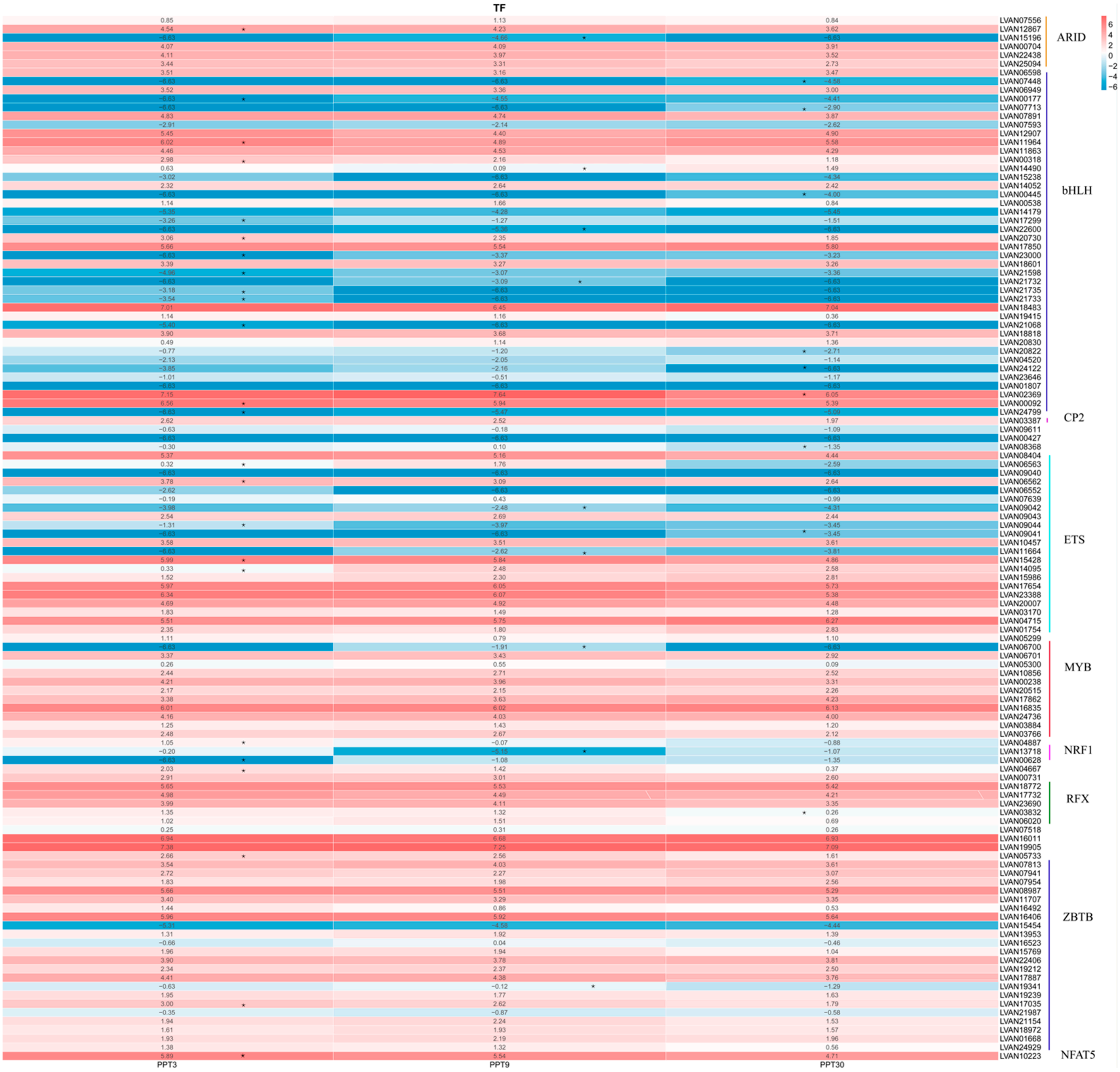

Fig. S12 Heatmap showing DEGs of encoding TFs. “*” indicates significant differential expression (foldchange > 2) of the *L. vannamei* among the salinities of 30‰ (control group), 9‰, and 3‰.

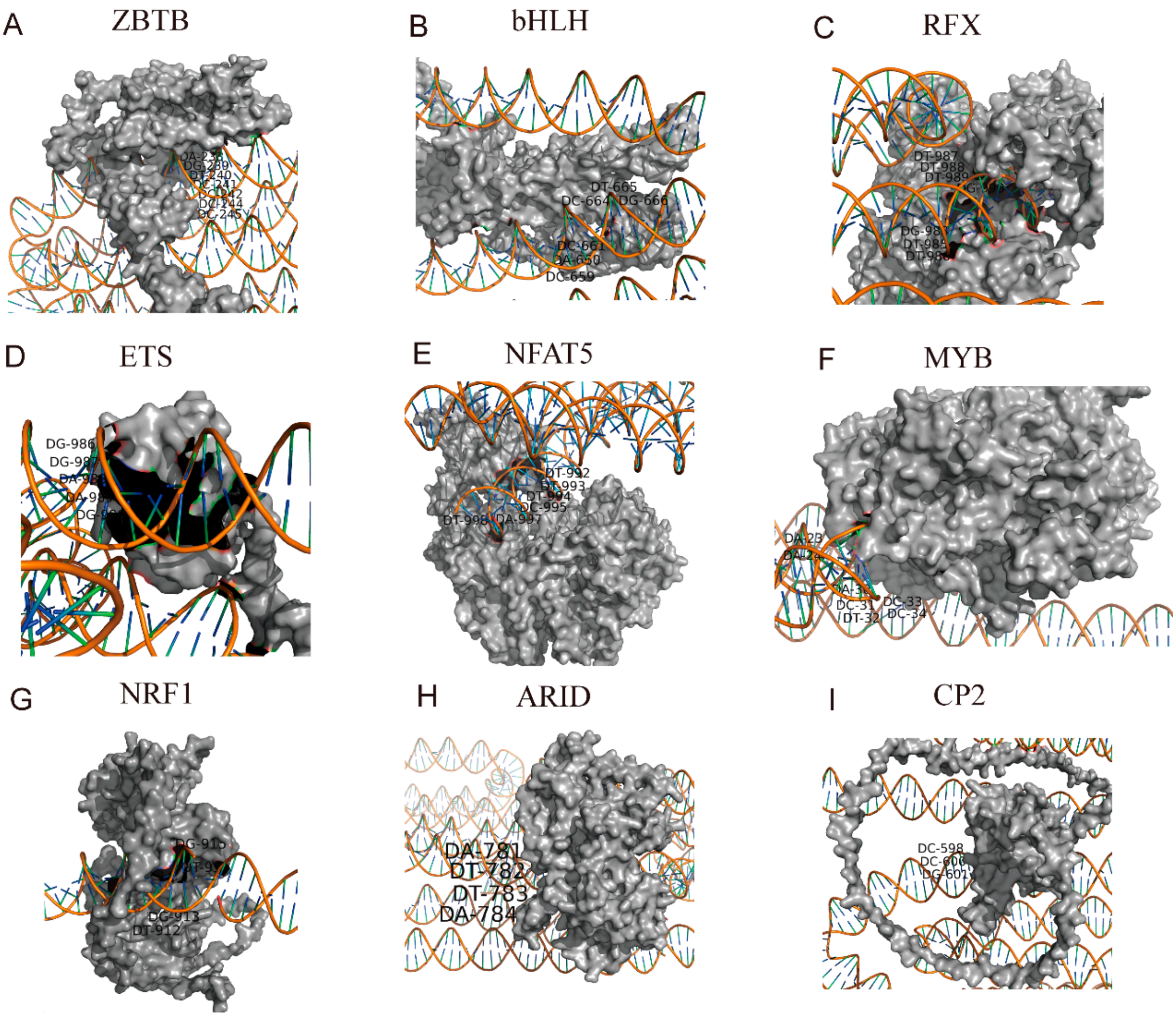

Fig. S13 Structural prediction of the complex of TFs and correspondent TFBSs for nine TFs using AlphaFold3. (A B C) Structural prediction of the complex of TFs and correspondent TFBSs for ZBTB, bHLH and RFX; (D E F) Structural prediction of the complex of TFs and correspondent TFBSs for ETS, NFAT5 and MYB; (G H I) Structural prediction of the complex of TFs and correspondent TFBSs for NRF1, ARID and CP2.

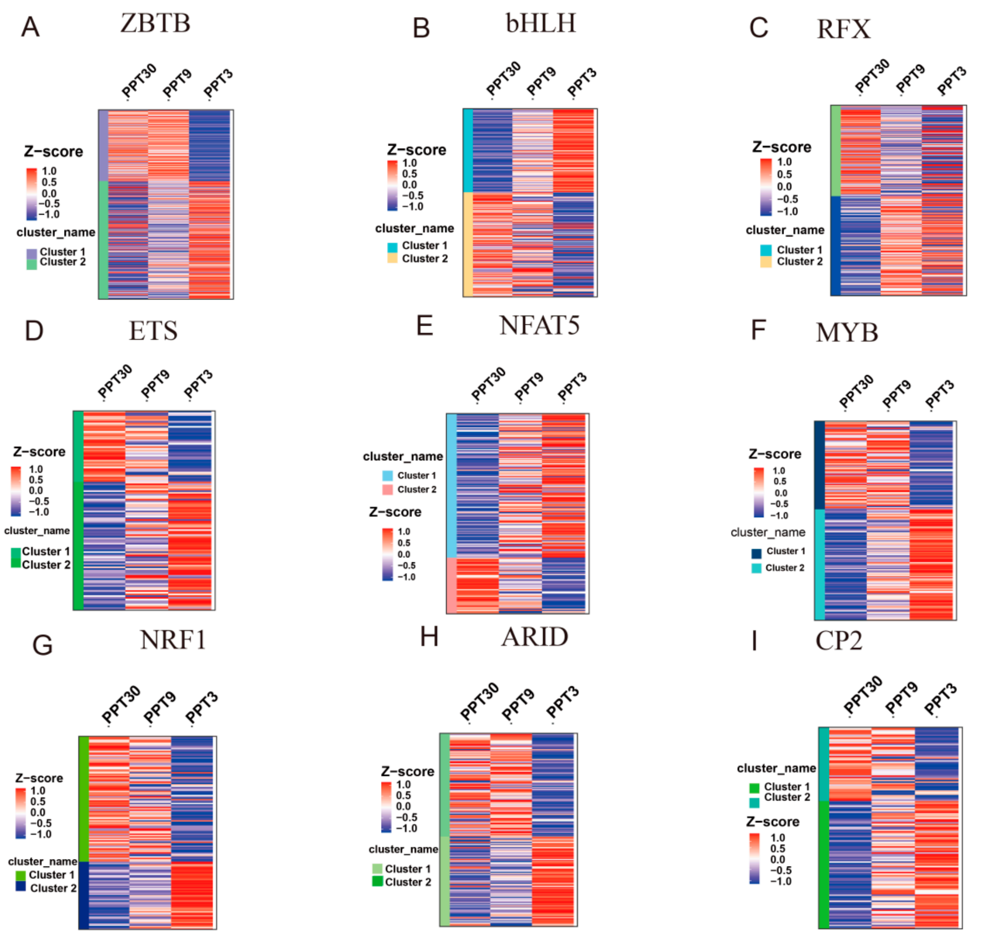

Fig. S14 Heatmap of targeted genes of the nine TFs in response to low salinity stress. (A B C) Heatmap of targeted genes of the ZBTB, bHLH, and RFX in response to low salinity stress; (D E F) Heatmap of targeted genes of the ETS, NFAT5, and MYB in response to low salinity stress; (G H I) Heatmap of targeted genes of the NRF1, ARID, and CP2 in response to low salinity stress.

Tables

Table S1 Summary of genes with various number of DPs.

Number of DPs	1DP	2DPs	3DPs	4DPs
Number of genes	4522	1003	147	19

Table S2 Summary of RNA-seq data.

Library Clean	sample number	Data(bp)	Q20(%)	Q30(%)
Lv30‰-1	3	6540180266	6356157872 (97.19%)	6060403044 (92.66%)
Lv30‰-2	3	7330527332	7135199179 (97.34%)	6823001829 (93.08%)
Lv30‰-3	3	9929173692	9646161607 (97.15%)	9128378844 (91.93%)
Lv9‰-1	3	6824579913	6616006093 (96.94%)	6242030064 (91.46%)
Lv9‰-2	3	7121769862	6912915227 (97.07%)	6580523625 (92.40%)
Lv9‰-3	3	8903596533	8596307550 (96.55%)	8070106756 (90.64%)
Lv3‰-1	3	7285650546	7069634057 (97.04%)	6724836569 (92.30%)
Lv3‰-2	3	6381343325	6188592202 (96.98%)	5840857071 (91.53%)
Lv3‰-3	3	6180832598	5992036453 (96.95%)	5652077168 (91.45%)

Table S3 Summary of ATAC-seq data.

Library Clean	sample number	Clean reads	Mapping reads	Mapping rate	peak number	Total length	Average length
Lv3‰-1	3	193377110	168,814,603	87.30%	300000	178,886,046	596.29
Lv30‰-1	3	247117160	188,863,104	76.43%	300000	191,071,458	636.9
Lv30‰-2	3	261584236	215,436,909	82.36%	300000	195,357,243	651.19
Lv3‰-2	3	345537552	300,198,312	86.88%	300000	149,052,689	496.84
